# Supplementary material for: Calculation of minimum energy pathways in transport proteins
Source: Commun Chem. 2025 Nov 25;8:377. doi: 10.1038/s42004-025-01754-1 (PMC12657958; doi:10.1038/s42004-025-01754-1)
Supplement: Supplementary file 2 — Supplementary Material PDF [file 42004_2025_1754_MOESM2_ESM.pdf]

Supplementary Information: Calculation of minimum energy pathways in transport proteins.

Briony A Yorke (1) & Helen M Ginn* (2,3)

(1) School of Chemistry, University of Leeds, Woodhouse Lane, Leeds, LS2 9JT, United Kingdom; (2) Center for Free-Electron Laser Science CFEL, Deutsches Elektronen-Synchrotron DESY, Notkestr. 85, 22607 Hamburg, Germany.
(3) Institute for Nanostructure and Solid State Physics; University of Hamburg; Hamburg 22761, Germany;
Corresponding author's email: helen.ginn@cfel.de;
Preprint: Preprint available on biorxiv **doi:** https://doi.org/10.1101/2024.08.07.607056. It is made available under a CC-BY-NC-ND 4.0 International license.

Classifications: Major- Biological Sciences, Minor - Biophysics and Computational Biology.

Keywords: Protein Dynamics, Conformational Transitions, Conformational Landscape, Computational Structural Biology.

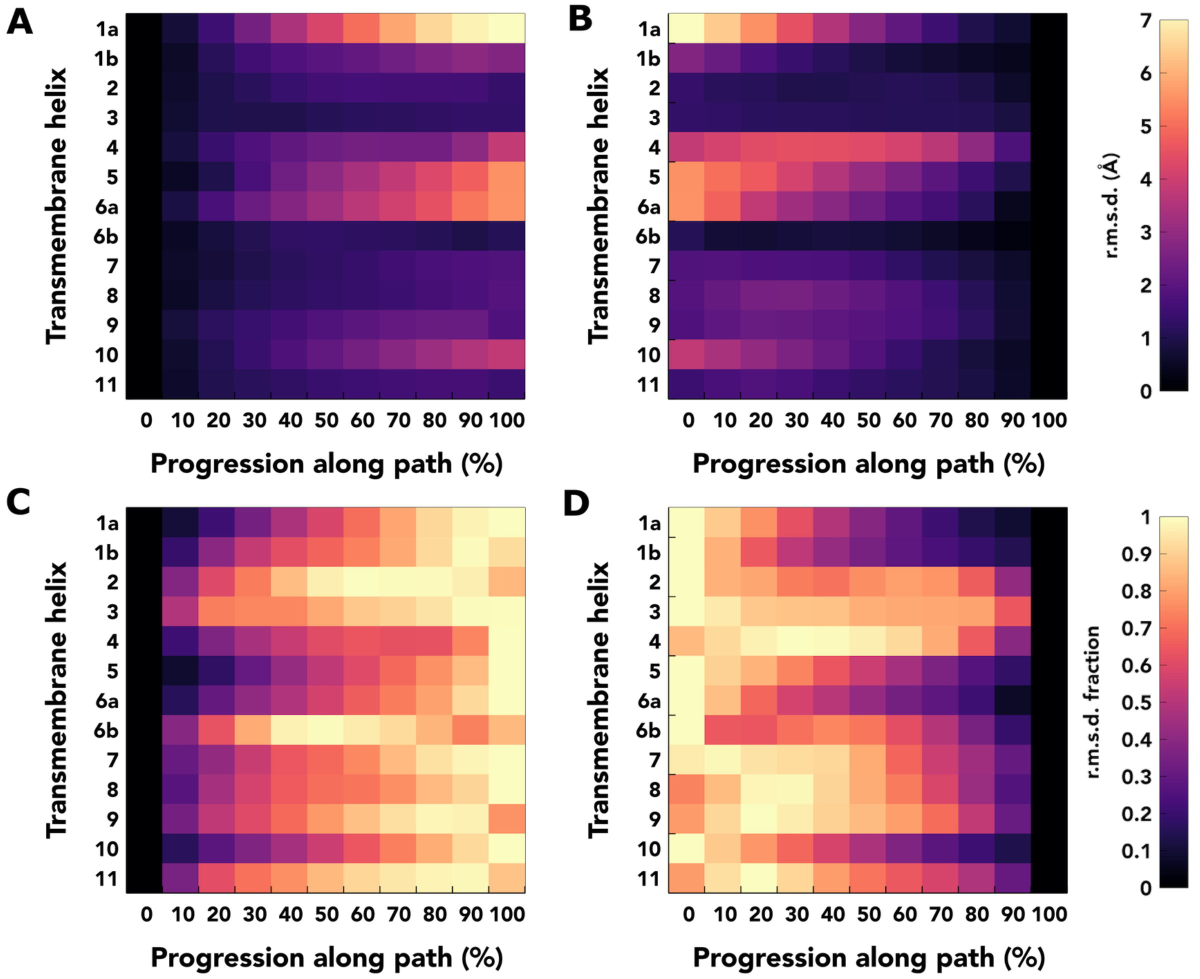

Supplementary Fig. 1: The r.m.s.d. of Cα atoms within each helix, showing the deviation of each helix (1 to 11) away from the start or end point of the DraNramp trajectory. (A, B) shows deviation in absolute r.m.s.d. after alignment of each mid-trajectory point against either (A) starting structure or (B) ending structure. As the midpoint at 0% is the same as the starting structure, and 100% is the same as the ending structure, the left column of (A) and the right column of (B) are all zeros. (C, D) shows an analogous alignment to (A, B) where each helix alignment has instead been expressed as a fraction of the maximum r.m.s.d. encountered along the pathway for that helix.

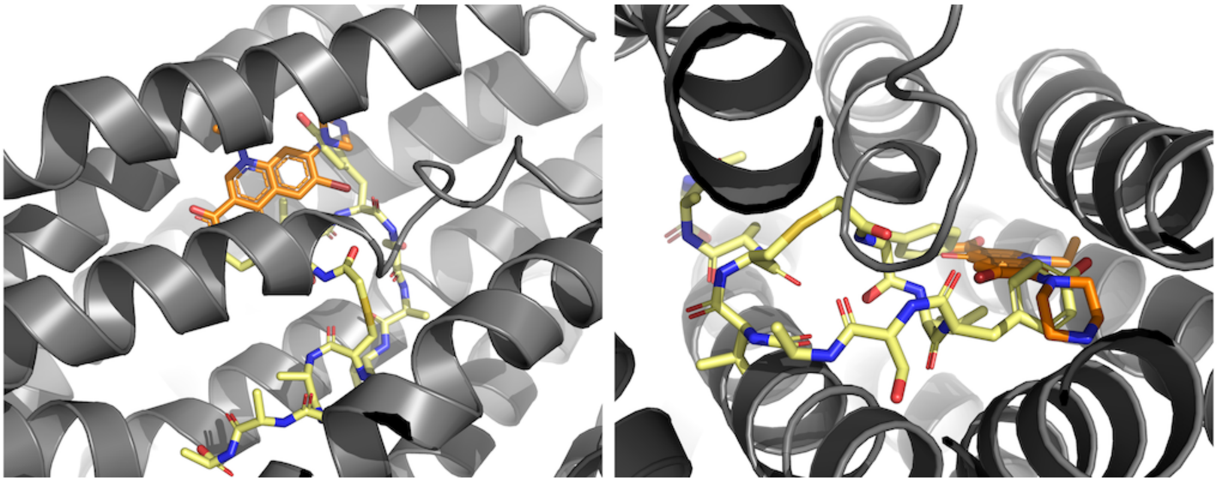

Supplementary Fig. 2: Two views of 70% progression (gray) through the MATE transition from inward- to outward-open, at the kinked TM1 unwinding position. Overlaid are the Br-NRF ligand-bound structure (orange, PDB: 3VVP) and the MaD5 inhibitor-bound structure (yellow, PDB: 3VVR) using all atoms in residues 165-185 for structure alignment (TM3, adjacent to ligand-binding site).

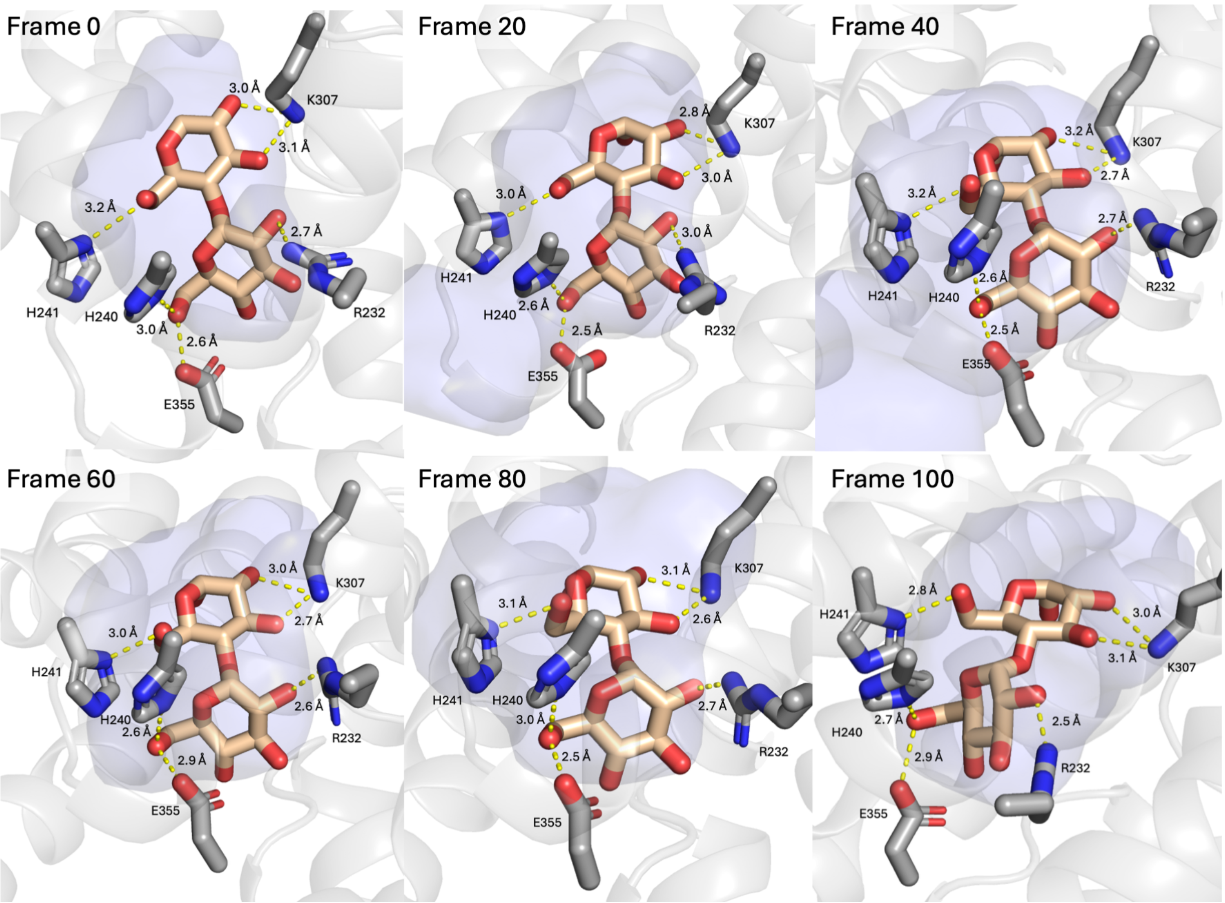

Supplementary Fig. 3: Key residues in the MalT (grey) binding cavity sampled at six equally spaced points along the pathway. Binding cavity shown as blue surface calculated using KVfinder [1]. Maltose ligand (yellow) taken from the ligand-bound structure was manually docked within the cavity to show hydrogen bonding is conserved throughout the pathway.

Supplementary Table 1: Alignment between start and end structures calculated using PyMOL [2], alongside time taken to establish the pathway on an MacBook Pro M2 using 8 threads and with manual clash resolution by an experienced user/developer. Backbone clashes remained in MATE, as highlighted in the text, where only the trajectory involving the straight helix was timed. Less experienced users are likely to require more time to generate trajectories.

**Name**	**Start:end structure r.m.s.d.** ($\AA^{-1}$)	**Processing time (mm:ss)**
MalT	7.50	06:59
DraNRamp	9.25	24:18
MATE (straight helix version)	5.77	34:04

Supplementary References

[1] J. V. Guerra, H. V. Ribeiro-Filho, J. G. Pereira, and P. S. Lopes-de Oliveira, "Kvfinder-web: a web-based application for detecting and characterizing biomolecular cavities," *Nucleic Acids Research*, vol. 51, no. W1, pp. W289–W297, 2023.

[2] W. L. DeLano *et al.*, "Pymol: An open-source molecular graphics tool," *CCP4 Newsl. Protein Crystallogr*, vol. 40, no. 1, pp. 82–92, 2002.
