# Supplementary material for: Calculation of minimum energy pathways in transport proteins
Source: Commun Chem. 2025 Nov 25;8:377. doi: 10.1038/s42004-025-01754-1 (PMC12657958; doi:10.1038/s42004-025-01754-1)
Supplement: Supplementary file 3 — Description of Additional Supplementary Files [file 42004_2025_1754_MOESM3_ESM.pdf]

Description of Additional Supplementary Files

File name: Supplementary Data 1

Description: 200 frame ensemble PDB file for the MalT trajectory.

File name: Supplementary Data 2

Description: 200 frame ensemble PDB file for the DraNramp trajectory.

File name: Supplementary Data 3

Description: 200 frame ensemble PDB file for the MATE trajectory.

File name: Supplementary Movie 1

Description: Video showing the calculated trajectory between the outward-open to inward-open structures of the EIIC domain of MalT . The N-terminal domain is coloured orange and C-terminal domain is coloured blue. The changes surrounding the helices AH2 and TM6 are highlighted.

File name: Supplementary Movie 2

Description: Video showing the calculated trajectory between the outward and inward-open structures of DraNramp. The changes observed around the Mn^{2+} binding site, TM6b and TM7 and the ligand binding site are highlighted.

File name: Supplementary Movie 3

Description: Video showing the calculated trajectory of the MATE transporter between the outward and inward-open structures. The TM1 kinked helix, ligand binding site (PDB:3VVP) and the effects of the inhibitor (PDB: 3VVR) are highlighted.
